# Supplementary figures and images for: 2,4-Diaminothieno[3,2-d]pyrimidines, a new class of anthelmintic with activity against adult and egg stages of whipworm
Source: PLoS Negl Trop Dis. 2018 Jul 11;12(7):e0006487. doi: 10.1371/journal.pntd.0006487 (PMC6062138; doi:10.1371/journal.pntd.0006487)

**OX02925 1H NMR**


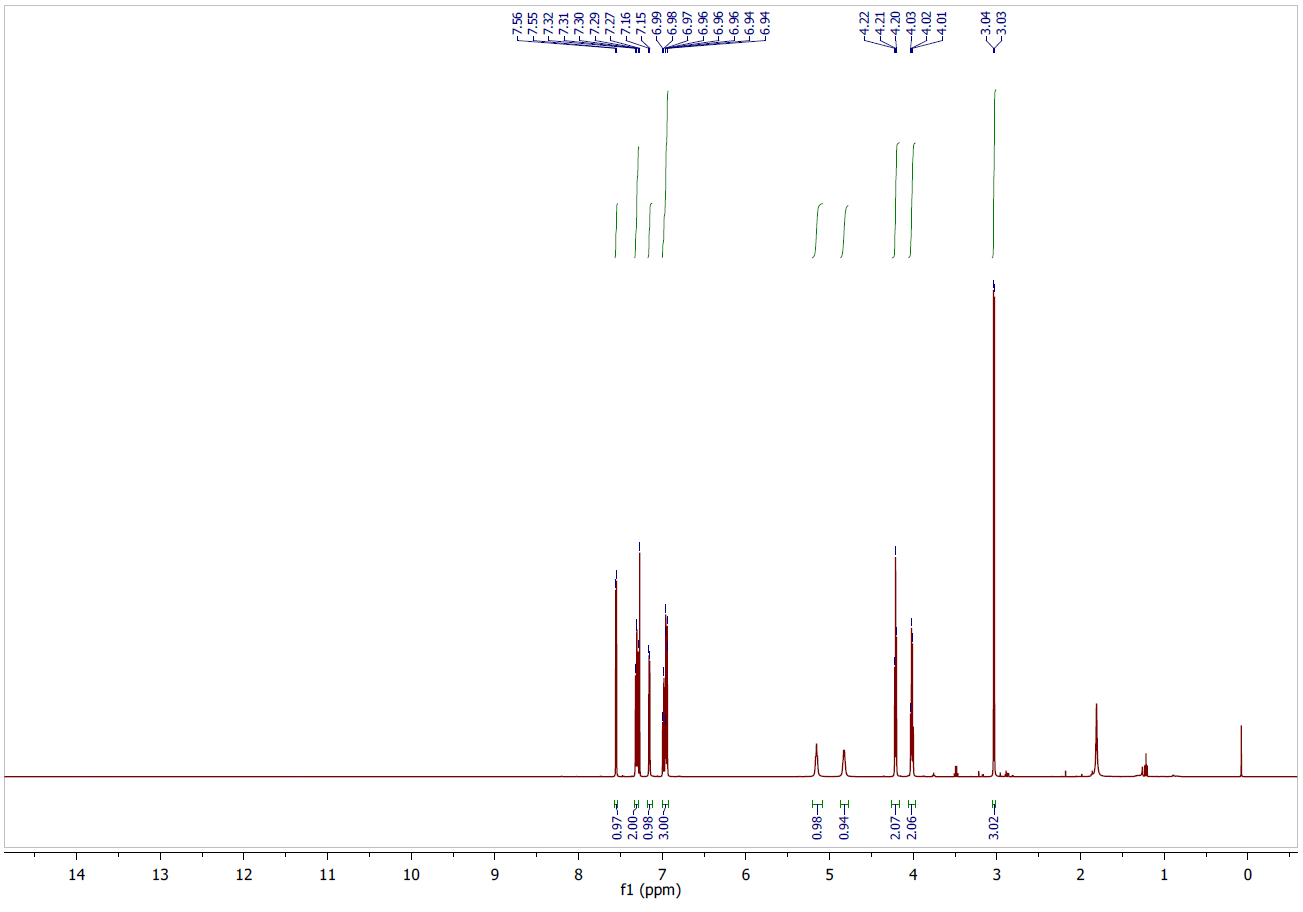


**OX02925 13C NMR**


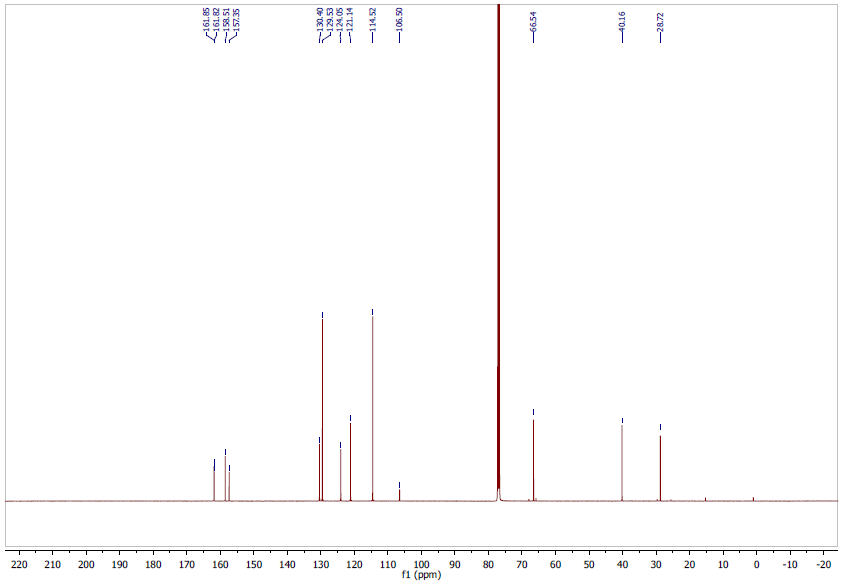


**OX02925 HRMS**


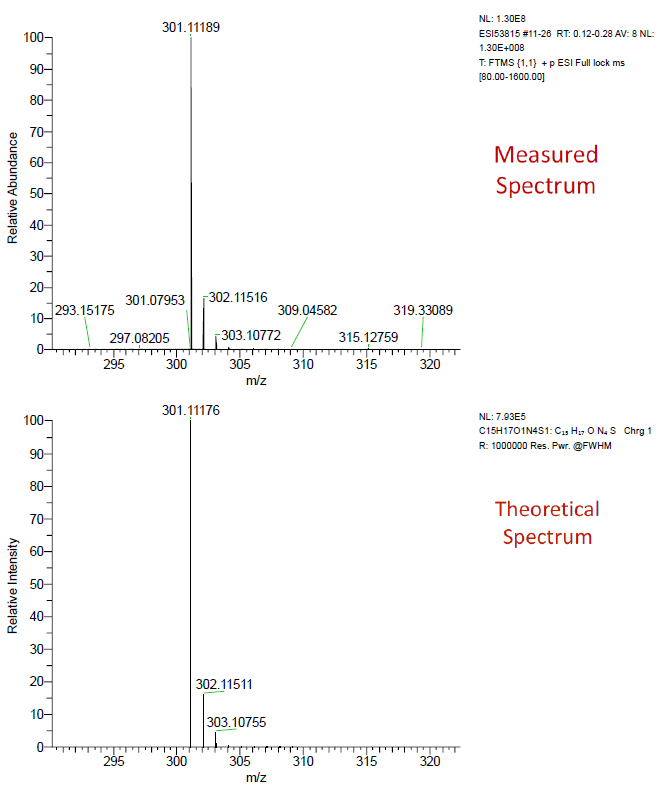


**OX02926 1H NMR**

**OX02926 13C NMR**

**OX02926 HRMS**


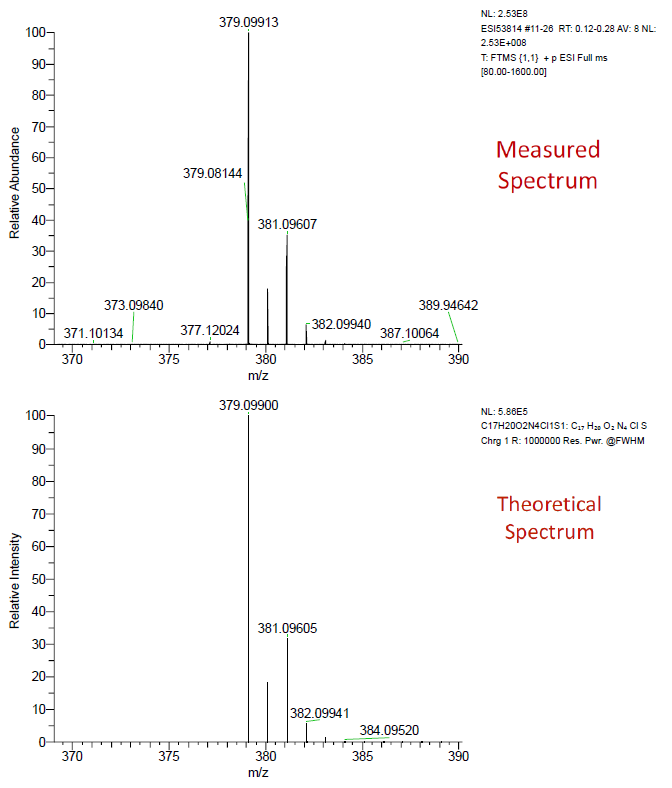


**OX03143 1H NMR**

**OX03143 13C NMR**

**OX03143 HRMS**


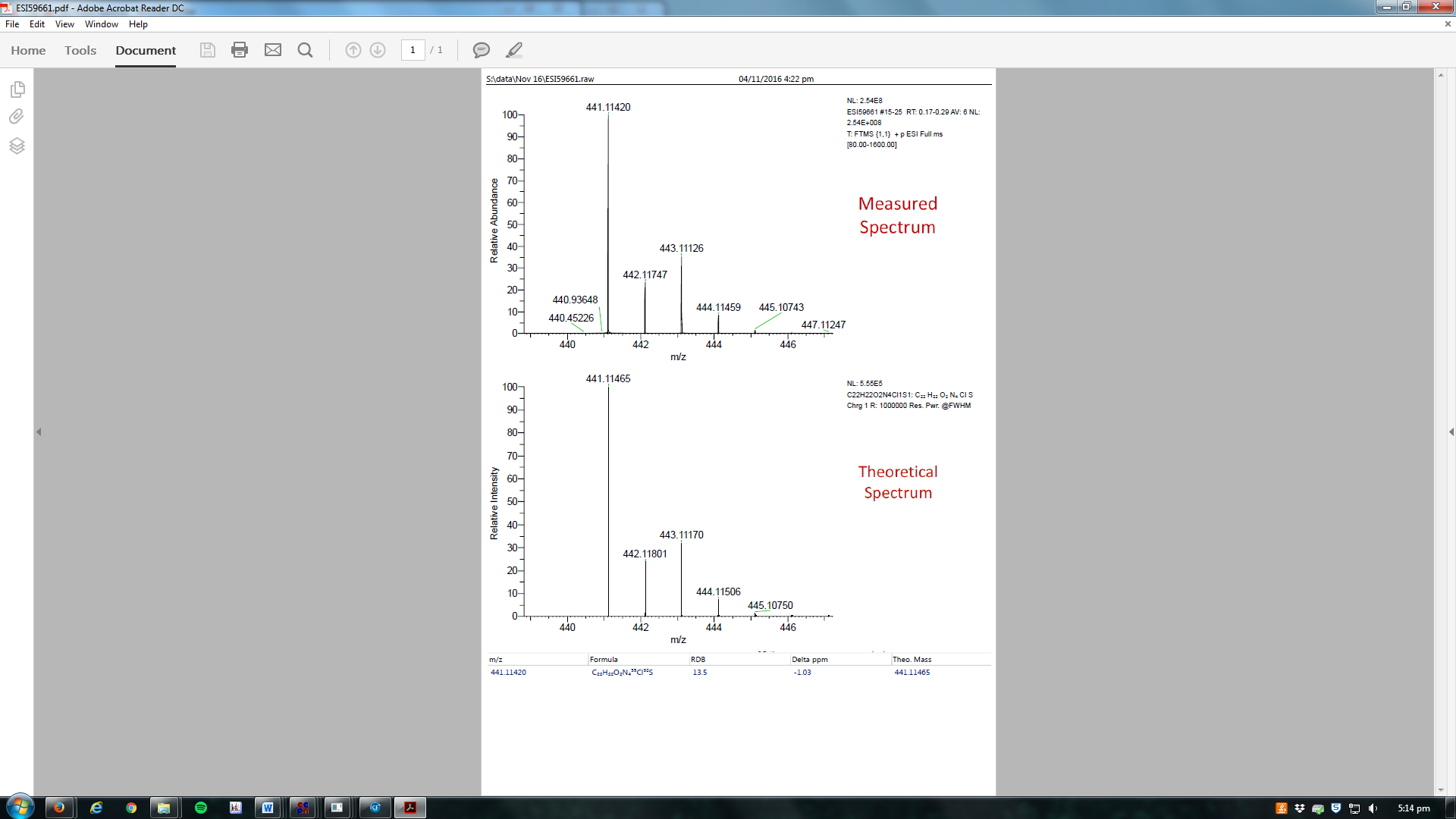


**OX03147 1H NMR**

**OX03147 1H NMR**

**OX03147 HRMS**


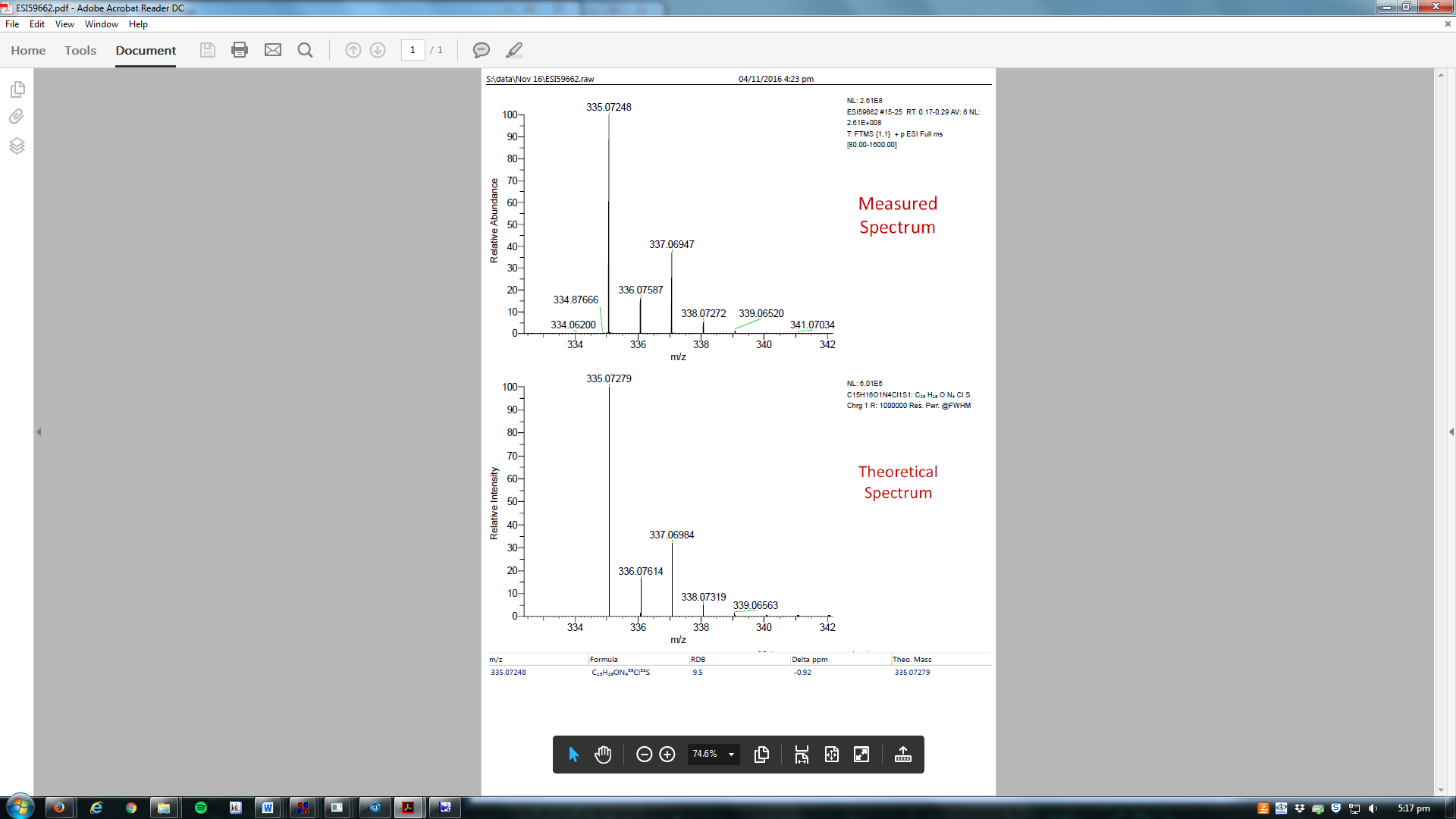

Supplement: S1 File — (DOCX) [file pntd.0006487.s001.docx]

Number of worms that established  
infection in each mouse

Batch: 1

20  
15  
10  
5  
0

DMSO

OX02926

treatment

Batch: 2

DMSO

OX02926

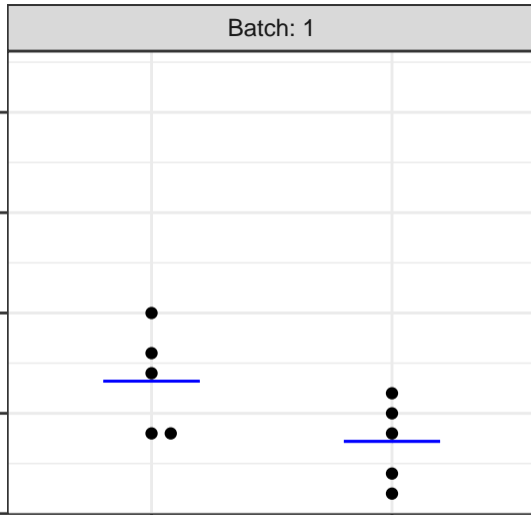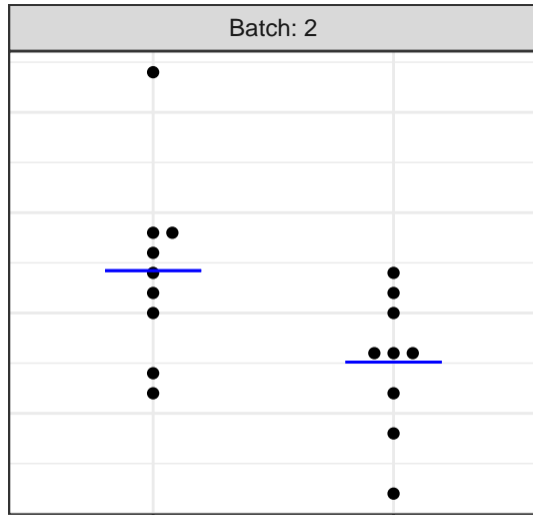

Supplement: S1 Fig — Each point indicates one mouse that has been infected with T. muris eggs that had been treated with deionised water plus 1% v/v DMSO (control) or deionised water plus 1% v/v DMSO and final concentration 100μM OX02926 for 14 days. Blue line indicates mean for each treatment group. (PDF) [file pntd.0006487.s002.pdf]

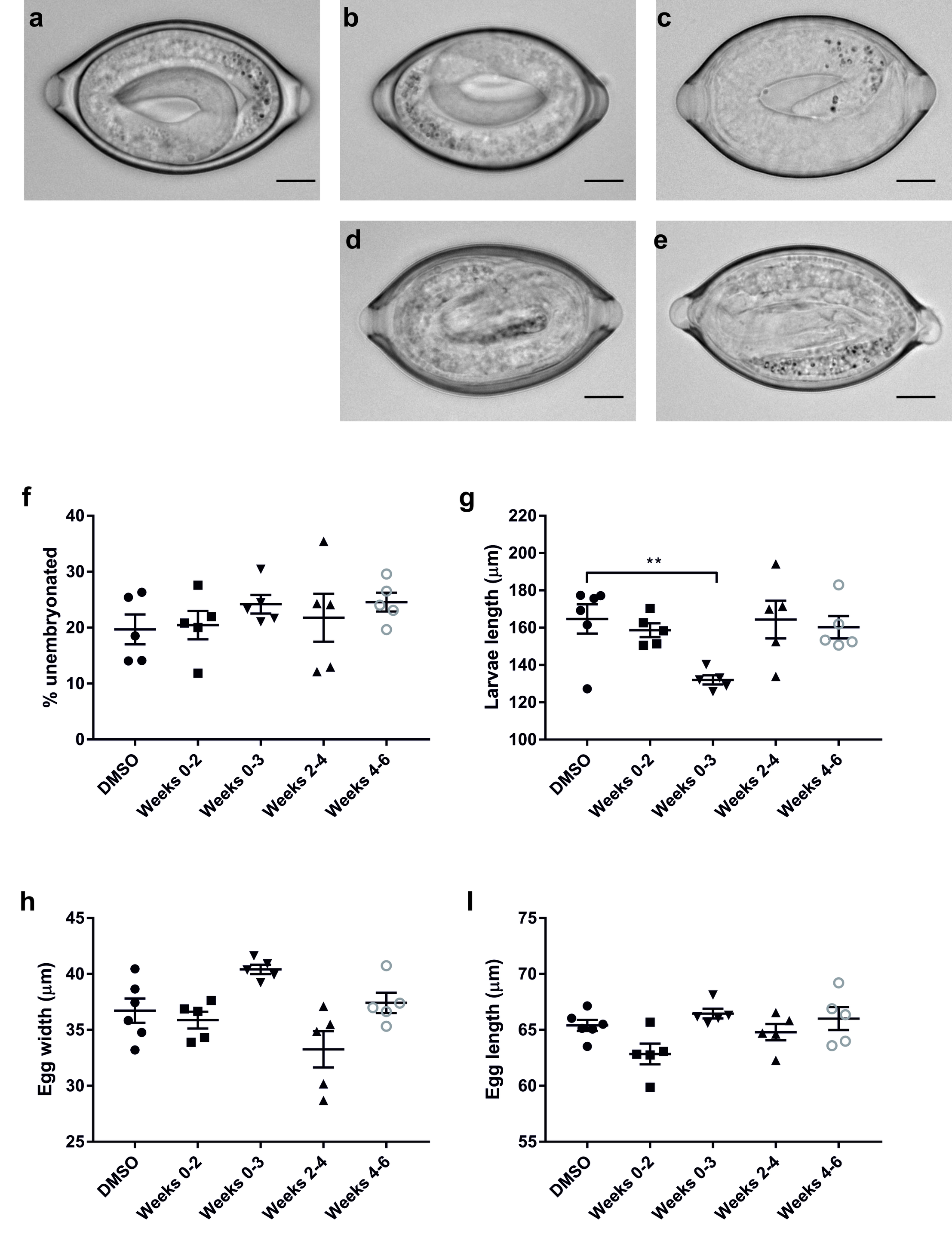

Supplement: S2 Fig — Unembryonated eggs were soaked in 100 μM OX03147 at 26°C for the duration specified and then embryonation determined and eggs imaged using an Olympus BX63 microscope. Scale bar indicates 10 μm. Representative pictures of (a) DMSO, (b) OX03147 weeks 0–2, (c) OX03147 weeks 0–3, (d) OX03147 weeks 2–4, (e) OX03147 weeks 4–6. Following 56 days embryonation was determined (f) and larvae length (g), egg width (h) and egg length (i) calculated using ImageJ. ** Indicates P < 0.01, one way ANOVA with post-hoc Dunnett’s test compared to DMSO control. (TIF) [file pntd.0006487.s003.tif]
