# Supplementary material for: 2,4-Diaminothieno[3,2-d]pyrimidines, a new class of anthelmintic with activity against adult and egg stages of whipworm
Source: PLoS Negl Trop Dis. 2018 Jul 11;12(7):e0006487. doi: 10.1371/journal.pntd.0006487 (PMC6062138; doi:10.1371/journal.pntd.0006487)
Supplement: S3 Fig — (PDF) [file pntd.0006487.s004.pdf]

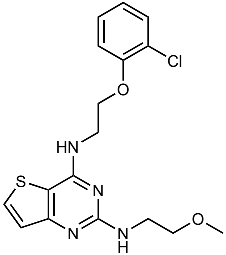

OX02926

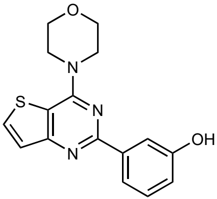

Hayakawa et al 2006

PI3K / mTOR inhibitor

PubChem CID: 9901372

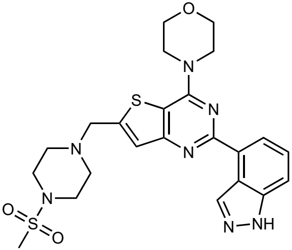

GDC-0941 (pictilisib)

PI3K / mTOR inhibitor

PubChem CID: 17755052

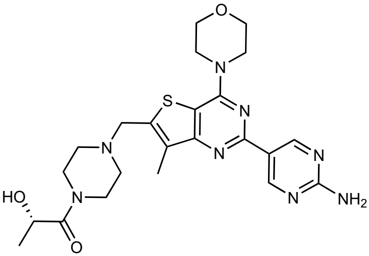

GDC-0980 (apitolisib)

PI3K / mTOR inhibitor

PubChem CID: 25254071

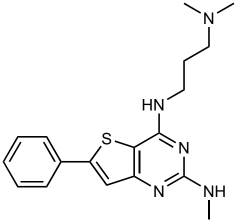

González Cabrera et al 2014

anti-malarial

PubChem CID: 76310732

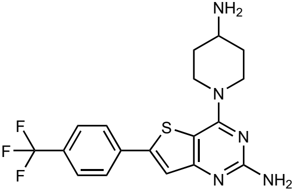

González Cabrera et al 2015

anti-malarial

PubChem CID: 122190278
